# Predictors of low trust in national healthcare systems in 30 countries

**DOI:** 10.1186/s12913-026-15026-8

**Published:** 2026-06-29

**Authors:** Andreas Heinz

**Affiliations:** Department of Health; Research Center for Public, Planetary and Digital Health, International University of Applied Sciences, Juri-Gagarin-Ring 152, 99084 Erfurt, Thuringia Germany

**Keywords:** Trust, Healthcare systems, International Social Survey Programme

## Abstract

**Background:**

Trust in national healthcare systems is associated with health behaviour and patient outcomes. Individuals with high trust are more likely to participate in preventive services and adhere to medical advice, whereas low trust is linked to poorer health, reduced service use, and lower patient satisfaction. Despite extensive research on interpersonal trust in healthcare professionals, cross-national evidence on public trust in healthcare systems remains limited. This study examines levels of trust in 30 healthcare systems and identifies sociodemographic, attitudinal and experiential predictors associated with low trust.

**Methods:**

The analysis draws on nationally representative data from the 2021 International Social Survey Programme, including 40,392 respondents aged 18 and older across 30 countries. Trust was dichotomised as high versus low trust. Multilevel logistic regression models with random intercepts at the country level were applied to account for data clustering. Individual level predictors included sociodemographic characteristics, health status, political orientation, attitudes toward vaccination and alternative medicine, perceptions of doctors, perceived inequalities in access, personal experiences with access barriers, and satisfaction with the last medical visit. At country level, the Corruption Perceptions Index was used.

**Results:**

Trust showed wide variation across countries, peaking in Scandinavian nations and remaining comparatively low in countries such as Poland, Suriname, and Russia. Logistic regression indicates that a high level of trust is associated with low levels of corruption at country level. Younger respondents and women were more likely to report low trust. Poor self-rated health emerged as one of the strongest predictors of low trust. Negative attitudes toward vaccination and voting for extreme political parties were also associated with low trust. Furthermore, both personal experiences of access barriers and perceptions of unequal access were associated with a higher likelihood of low trust. Respondents dissatisfied with their last medical treatment and those with generally negative views of doctors showed markedly lower trust in the healthcare system.

**Conclusion:**

Trust in national healthcare systems is associated with a broad set of individual and contextual factors. These findings highlight multiple potential leverage points for strengthening trust, ranging from improving doctor–patient interactions to addressing structural inequalities in healthcare access.

**Supplementary Information:**

The online version contains supplementary material available at 10.1186/s12913-026-15026-8.

## Background

Multiple studies have shown that low trust in the healthcare system is associated with risky health behaviours and worse health outcomes. For example, Mexicans and Americans who do not trust the healthcare system are significantly more likely to use antibiotics without a prescription than those who do trust it [1]. A review on breast cancer screening and treatment reported that low trust in the healthcare system is linked to lower participation in screening, less use of healthcare services, and reduced patient satisfaction and quality of life [2]. It is therefore important to identify which factors predict trust in the healthcare system. This study addresses this question using data from the International Social Survey Programme (ISSP). The next section defines trust and explains its relevance for health. It then outlines the theoretical approaches and empirical studies that provide an explanation for trust in the healthcare system. The introduction concludes with a summary of these perspectives to justify the specific predictors of trust examined in this study.

### Definition and relevance of trust

Although trust can be defined and measured in various ways [3], most definitions share a common core. Following Bi et al. [4], trust is understood as a psychological state of an individual (the trustor) directed toward a specific target (the trustee), which may be a person (e.g., a physician) or an abstract entity (e.g., the national healthcare system). The crucial aspect is that the trustor depends, at least in part, on the trustee to achieve their goals. From a sociological and economic perspective, trust involves voluntarily accepting vulnerability in expectation of a benefit [5, 6], for example, by paying into a health insurance scheme today, trusting that it will help you when you need it and that the money will not be misappropriated. When the trustee is a social institution or system, this is referred to as public trust; when the trustee is another person, it is referred to as interpersonal trust [7]. The Model of Public Trust in Healthcare proposes that these two forms of trust interact. For example, positive personal experiences with physicians can enhance trust in the healthcare system as a whole [8]. In empirical research, however, the emphasis is more often on interpersonal trust in healthcare professionals than on trust in the healthcare system overall [9, 10].

A meta-analysis of cross-sectional and longitudinal studies found a positive correlation between trust and well-being (*r* = 0.21) [4]. The analysis included both public trust and interpersonal trust. Among the longitudinal studies, 55 indicated that trust predicts later well-being, and 49 showed that well-being predicts later trust, suggesting a mutual reinforcing relationship between the two.

Trust in institutions has been examined extensively, especially during the COVID-19 pandemic. In the early months of the pandemic, European countries with higher levels of trust in state institutions (such as parliament, politicians, political parties, the police, and the judiciary) had lower mortality rates [11]. Subsequent research has focused more specifically on trust in the healthcare system. An online survey from Norway found that lack of trust in the healthcare system functioned as a moderator [12]: at that time, certain population groups experienced greater psychological distress during the COVID-19 pandemic when their trust in the health system was low. In addition, a review showed that low trust in the healthcare system predicts a lower willingness to be vaccinated against COVID-19 [13].

The relationship between trust and willingness to be vaccinated was already being investigated before the COVID-19 pandemic. A qualitative study showed that white and African American adults differ substantially in how much they trust government statements about influenza vaccination, and that this trust strongly influences their willingness to be vaccinated [14]. These differences were linked, among other factors, to historical experiences such as the Tuskegee study.

### Theories on the origins of trust and known predictors of trust

Trust is a key concept in several social science disciplines. As a result, there are multiple theoretical approaches to explaining trust in the healthcare system, but no single framework that integrates all of them. The following section therefore outlines different perspectives on trust and its determinants.

Trust can be understood as a relatively stable personality trait that develops, for example, through socialisation [4]. From this perspective, trust is general rather than strongly dependent on specific situations, so people who tend to be trusting overall are also more likely to trust the healthcare system. This view was supported by a panel study by Busemeyer [15] conducted during the COVID-19 pandemic: individuals with high general political trust also had high confidence that they would receive necessary medical care. Other studies likewise show that trust in different social institutions is correlated [16, 17]. In line with this, early studies already found a positive association between how people evaluate the national medical profession and their trust in the healthcare system [7, 18]. The idea that trust is a stable trait acquired through socialisation may also help explain differences in trust between ethnic groups [2, 14, 19] and between countries [10, 16].

However, trust can also be seen as the outcome of previous experiences [4, 5]. In this view, perceived barriers to access [20] as well as personal experiences of such barriers, are known predictors of low trust in the healthcare system [10]. Nevertheless, the two perspectives—trust as a stable personality trait and trust as a result of experience—are not mutually exclusive [4].

A third psychological approach is mood congruence, which proposes that a positive or negative mood shapes how people perceive and evaluate other aspects of the world, leading them to judge these more positively or negatively. This helps explain the association between well-being and trust mentioned above [4]. In line with this, studies have found that trust in the healthcare system is lower among people who rate their own health as poor [21, 22] and among those with chronic illnesses [10].

Another predictor of trust is political orientation. A study from Slovenia found that trust in the healthcare system is higher among voters of left-wing parties than among right-wing voters [23]. The authors explain this by suggesting that right-wing voters tend to be more sceptical of scientific explanations and of information provided by medical professionals. Empirically, the study showed that right-wing voters are more likely to favour alternative medicine, and that this preference is associated with lower trust in the healthcare system. This and other studies have also found a positive association between trust in the healthcare system and attitudes towards vaccination, including the intention to be vaccinated [14, 23].

From a socio-economic perspective, social stratification can help explain levels of trust. Individuals who belong to groups that generally feel disadvantaged or treated unfairly by social institutions may extend this perception to the healthcare system [9]. Consistent with this idea, several studies have reported a positive association between higher educational attainment and higher trust in the healthcare system [10, 21], while another study found a negative association [24].

Findings for other sociodemographic characteristics are also inconsistent. In the study by Zhao et al., older age was associated with higher trust [22], whereas Lamot et al. found the opposite pattern [23]. In contrast, Nikodem et al. reported no significant age effect [17]. Results for gender are similarly mixed: Kruk et al. found lower trust in the healthcare system among women [10], Chen and Cheng observed lower trust among men [20], and Nikodem et al. again found no effect [17].

The preceding paragraphs have shown that, in theory, an individual’s trust is shaped by the individual’s characteristics and experiences, as well as by their close social environment during socialization. Sociological and political science theories further emphasize the role of society in the formation of trust, with the concept of social capital playing an important role. In societies where people are strongly connected and trust one another, social capital is high, which enables these societies to function better because cooperation is rewarded and transaction costs are lower [25]. From a temporal perspective, it is important to note that cooperation and trust can reinforce each other when successful, or lead to a downward spiral in the case of failure [26]. Empirical evidence from numerous studies over recent years shows that generalized trust is highest in Scandinavia [26–28]. A country’s social capital can continue to grow in societies where political institutions make decisions and act fairly and impartially, which leads to the corruption-trust theory [29]. In brief, this theory states that people use the behaviour of public officials as a heuristic for judging whether people in this society can be trusted. If public officials habitually abuse their power for private gain, this contributes to a decline in trust [30]. Empirical findings have shown that in countries that became less corrupt over time, trust increased [31]. Using cross-sectional data from 120 countries, Holmberg and Rothstein were able to show that corruption is associated with lower life expectancy and poorer subjective health [32].

### The present study

In summary, trust in the healthcare system can be explained by several theoretical approaches. Empirical studies have identified multiple predictors of trust, although findings are sometimes inconsistent, especially for sociodemographic factors. Another limitation in the existing literature is that many studies are restricted to single countries. This study therefore analyses trust in the healthcare systems of 30 countries, examining the following predictors: sociodemographic differences (age, gender, educational level), health status (self-rated health and chronic conditions), political attitudes and attitudes towards vaccinations and alternative medicine, perceptions of doctors in general and personal experiences with doctors as key representatives of the healthcare system. In addition, perceptions of barriers to access and personal experiences with such barriers are analysed as predictors of trust. The analysis also examines differences between countries and the extent to which these can be explained by corruption.

## Methods

### ISSP data

The data are drawn from the 2021 International Social Survey Programme (ISSP) Health and Health Care module. The questionnaire was developed by an ISSP drafting group and approved by the ISSP General Assembly, which also assesses its ethical appropriateness. Principal investigators in each participating country are responsible for ensuring compliance with national legal requirements [33]. In total, 30 countries took part in ISSP 2021. The target population generally included individuals aged 18 years and older, although some countries also surveyed younger respondents and applied upper age limits (e.g., Finland: 15–74 years). Data collection was carried out using face-to-face interviews (paper-and-pencil or computer-assisted), self-completed questionnaires (paper or web-based), or telephone interviews. Fieldwork started on 5 February 2021 and ended on 30 April 2024. The international dataset comprises 44,549 respondents, with national sample sizes ranging from 1,001 in Mexico to 3,349 in Switzerland. The data are available after free registration. Further details on survey methodology are documented in the GESIS Variable Report [34].

### Measures

Trust was measured with the question “In general, how much confidence do you have in the health care system in [country]”. For the logistic regression analysis, the response categories “complete confidence” and “a great deal of confidence” were combined into “high trust”, while “some confidence,” “very little confidence,” and “no confidence at all” were combined into “low trust.” This cut-off point has previously been used in studies based on the ISSP 2011 [22, 24].

Sociodemographic information included gender (male/female) and year of birth, which was used to calculate age. Respondents reported their highest educational qualification, which was then grouped into three categories (low, middle, high) based on the International Standard Classification of Education [35].

Health status was assessed with two questions. First, self-rated health was measured on a five-point scale [36]. Second, respondents were asked whether they had a chronic illness or disability, with response options “yes” or “no.”

Attitudes toward alternative medicine were assessed with the item: “Alternative medicine provides better solutions for health problems than mainstream medicine,” answered on a five-point Likert scale. Attitudes toward vaccination were measured using two critical statements about vaccines; the mean of these two items was calculated (1 = positive attitude, 5 = negative attitude).

Political attitudes were derived from reported voting behaviour in the most recent national election. The ISSP research group classified all reported parties into five left–right categories. For this analysis, an additional category was created that includes non-voters, respondents who did not answer the question, and those who reported a party that could not be placed on the left–right spectrum.

Perceived inequalities in access to healthcare were measured by asking respondents whether they believe access to healthcare is easier or harder for certain social groups (e.g., rich vs. poor). Responses ranged from 1 “much easier” through 3 “about the same” (neutral) to 5 “much harder.” For four such items, deviations from the neutral category were summed, yielding a score from 0 (equal access for all groups) to 8 (maximum perceived inequality).

In addition to perceptions of inequality, respondents were asked about their own experiences with access barriers. They were asked whether, in the past year, they had not received needed treatment for any of three reasons (could not afford it; no time/other commitments; waiting list). Reporting at least one of these reasons was classified as having personal experience of access barriers.

General trust in doctors was measured with three items capturing perceptions of doctors’ competence, fidelity, and overall trust, based on Hall et al. [37]. For the analyses, the mean score was computed so that evaluations of doctors in the respondent’s country ranged from 0 (very positive) to 5 (very negative).

Respondents were also asked how satisfied they were with the treatment received at their last medical visit, using a seven-point scale from completely satisfied to completely dissatisfied.

For all multi-item measures, scale scores were calculated using available valid responses to reduce loss of cases due to missing data. Non-substantive responses (e.g., “not applicable”) were assigned to neutral categories.

Corruption was measured using the Corruption Perceptions Index (CPI) 2021, which is published annually by Transparency International. This is a composite indicator at country level that measures perceived corruption in the public sector and draws on 13 data sources [38].

### Analysis

Country differences in trust in the national healthcare system were calculated as percentages and displayed in a stacked bar chart. In this and all subsequent analyses, a combined weight was applied to account for different selection probabilities within countries.

To identify predictors of low trust in the healthcare system, a multilevel logistic regression was conducted using the GENLINMIXED procedure in IBM SPSS 24. A random intercept at the country level was included to account for the hierarchical data structure. For sensitivity analysis, models with random slopes were also estimated, but these did not differ substantially from the random-intercept models. As random slopes are not central to the research questions and there is no strong theoretical justification for their use, only the random-intercept models are reported [39].

First, an empty model without predictors was estimated to assess potential clustering by country [40]. In Model 2, sociodemographic variables were added. Model 3 additionally included health status variables. Model 4 then incorporated attitudes toward alternative medicine and vaccinations, as well as voting behaviour. The final Model 5 further added CPI, perceptions of doctors and access to the healthcare system, along with experiences from the last doctor visit and personal experiences of access barriers.

To assess the robustness of the results, the multilevel analysis was repeated in two additional variants. The first variant examined whether the choice of cut-off value for the outcome influenced the findings. Rather than applying a dichotomous cut-off, an ordered logistic regression was carried out using all five outcome categories. The second variant assessed whether assigning non-substantive responses to the neutral response categories affected the results. For this purpose, non-substantive responses were excluded from the logistic regression. The findings of both analyses are presented in the supplementary material.

## Results

The original dataset includes 44,549 respondents from 30 countries (see Table 1). To ensure a consistent minimum age across countries, 110 respondents younger than 18 years were excluded. An additional 4,047 respondents were excluded because they had missing values on at least one variable used in the full model. The final analytic sample therefore consists of 40,392 respondents from 30 countries. The supplementary material includes a comparison between the original dataset and the analytic sample, showing only minor differences in the outcome and the independent variables.

**Table 1:** Sample exclusion flow for analytic dataset

Step	Description	Cases excluded	Remaining *N* (unweighted)
1	ISSP 2021 Health and Health Care	-	44,549
2	Exclusion of respondents younger than 18	110	44,439
3	Exclusion of respondents with missing values on at least one predictor or the dependent variable	4,047	40,392

**Fig. 1:**
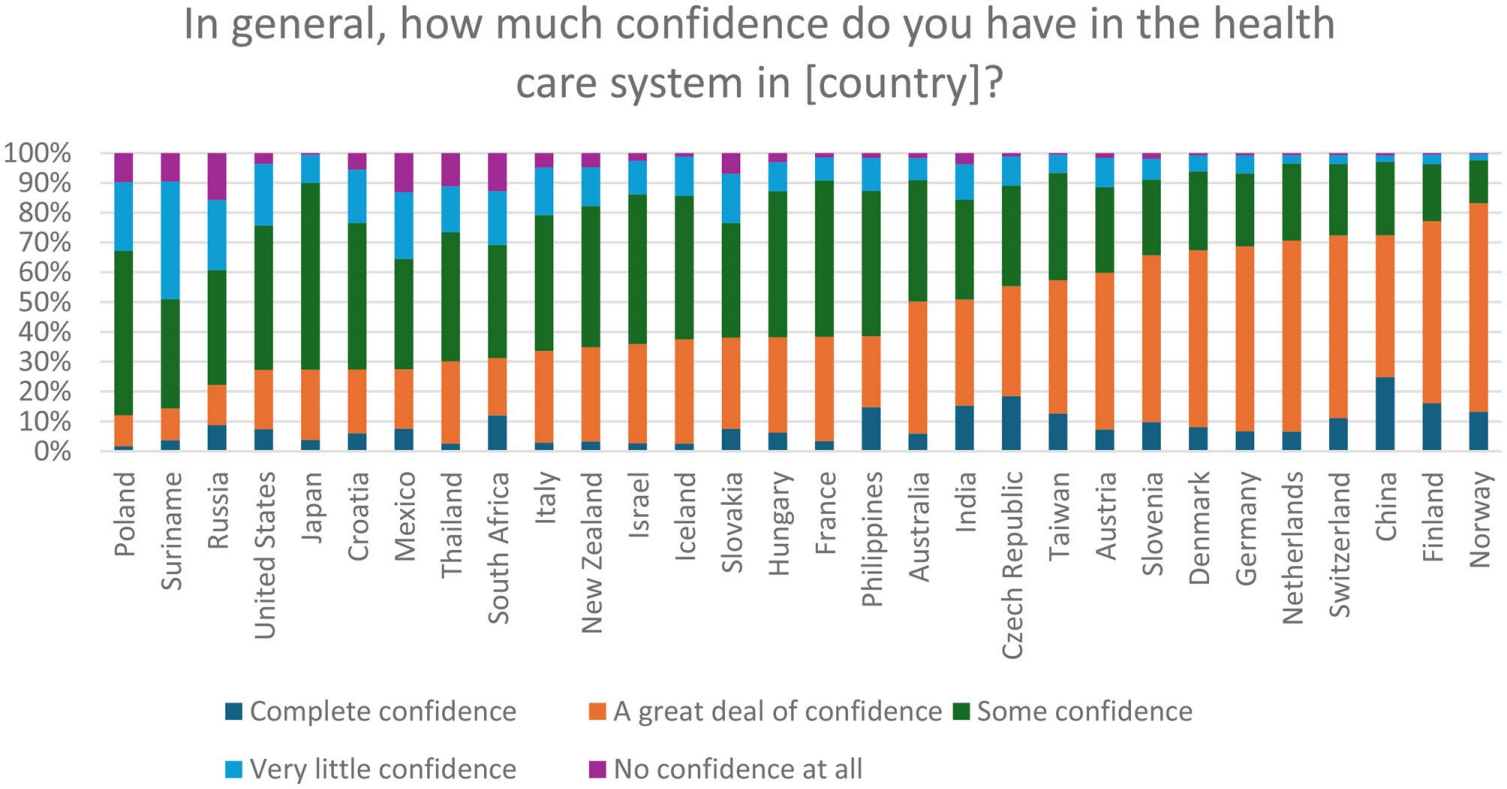
Trust in healthcare system by countries

Figure 1 displays levels of trust in national healthcare systems by country. Countries are ordered by the combined proportion in the two highest trust categories. At the top are mainly Scandinavian countries, as well as China, Germany, and several of Germany’s neighbouring countries. Countries with comparatively low trust in their healthcare systems are geographically dispersed, including Poland, Suriname, Russia, the United States, and Japan.

Table 2 presents the logistic regression results. In the null model without predictors, the intraclass correlation coefficient (ICC) is 0.197, which exceeds the 0.05 threshold and indicates that a multilevel approach is appropriate due to clustering of low trust within countries [41]. In Model 2, which includes sociodemographic variables, younger respondents and women are more likely to report low trust in the healthcare system; these associations remain stable in subsequent models. Respondents with low and medium educational attainment show lower trust than those with high education. For respondents with a low level of education, the association is no longer significant when health status is taken into account (Model 3), and the association reverses in the last two models.

**Table 2:** Multilevel logistic regression of predictors of low trust in the healthcare system

	Model 1: Null model with cluster effect	Model 2: Covariates added	Model 3: Health status added	Model 4: Medical scepticism and political orientation added	Model 5: Perception and experiences with doctors and access barriers and CPI added
Fixed Effects		OR (SE)	OR (SE)	OR (SE)	OR (SE)
Age		0.994 (0.001)***	0.990 (0.001)***	0.993 (0.001)***	0.996 (0.001)***
Gender (female; ref. = male)		1.082 (0.022)***	1.062 (0.022)**	1.062 (0.022)**	1.051 (0.024)*
Educational level (ref. = high)					
medium		1.214 (0.027)***	1.149 (0.027)***	1.039 (0.027)n.s.	0.992 (0.029)n.s.
low		1.129 (0.032)***	1.035 (0.033) n.s.	0.890 (0.033)***	0.819 (0.035)***
Self rated health (ref. = very good/excellent)					
good			1.612 (0.027) ***	1.615 (0.027)***	1.509 (0.029)***
poor/fair			2.470 (0.032)***	2.453 (0.033)***	2.026 (0.035)***
No chronic condition (ref. = chronic condition)			1.057 (0.028)*	1.034 (0.028) n.s.	1.074 (0.030)*
Alternative medicine better (ref. = agree)					
neutral or no answer				1.044 (0.032) n.s.	1.185 (0.034)***
disagree				0.806 (0.033)***	1.003 (0.034) n.s.
Attitude towards vaccination (1 = positive; 5 = negative)				1.335 (0.012)***	1.160 (0.013)***
Vote last election (ref. = center)					
no vote, no answer, other				1.286 (0.040)***	1.237 (0.042)***
Far right				1.629 (0.078)***	1.444 (0.083)***
Right, conservative				0.944 (0.046) n.s.	0.961 (0.048) n.s.
Left, center				0.938 (0.047) n.s.	0.959 (0.049) n.s.
far left				1.206 (0.075)*	1.212 (0.079)*
Experienced barriers to access in the healthcare system (yes; ref. = no such experience)					1.307 (0.030)***
Perception of equality of access to healthcare in country (0 = equal; 8 = highly unequal)					1.037 (0.007)***
Perception of doctors in country (0 = positive; 5 = negative)					2.324 (0.018)***
Satisfied with last treatment by a doctor (ref. = satisfied)					
neutral or no answer					1.961 (0.035)***
not satisfied					2.361 (0.052)***
CPI (0 = corrupt; 100 = clean)					0.985 (0.008)*
**Random effects**					
Variance explained at country level (SE)	0.805 (0.213)	0.789 (0.209)	0.789 (0.209)	0.732 (0.194)	0.624 (0.168)
**ICC Country**	0.197	0.193	0.193	0.182	0.159
**Model fit**					
AIC	185480,367	185700,589	186879,434	188420,037	195157,944
BIC	185488,973	185709,196	186888,041	188428,643	195166,550
**Sample sizes**					
n (participants)	40,392	40,392	40,392	40,392	40,392
n (countries)	30	30	30	30	30

***p < 0.001; **p < 0.01; *p < 0.05. AIC, Akaike Information Criterion; BIC, Bayesian Information Criterion; CPI, Corruption Perception Index; OR, Odds Ratio; ref., reference category; SE, Standard error of the log-odds

Self-rated health is a strong predictor: the worse respondents rate their health, the higher their probability of low trust in the healthcare system. By contrast, having a chronic disease is not a consistent predictor. In Model 3, respondents without chronic conditions are slightly more likely to have low trust, whereby the relationship is no longer significant in Model 4 but becomes significant again in Model 5.

Attitudes toward alternative medicine also show no consistent effect. In Model 4, respondents who disagree that alternative medicine is better than mainstream medicine are less likely to have low trust than those who prefer alternative medicine, but this association disappears in Model 5. In contrast, a negative attitude toward vaccination is a clear and stable predictor of low trust in the healthcare system. Voting behaviour shows another clear pattern, using centrist voters as the reference category. Voters for moderate parties to the left or right of centre do not differ significantly from the reference group. However, voters for extreme right-wing and extreme left-wing parties, as well as non-voters, are more likely to report low trust in the healthcare system.

The full Model 5 demonstrates that both doctors and access barriers are important for trust. Personal experience of barriers to access is associated with higher odds of low trust. Independently of personal experience, perceiving that access to healthcare is easier or harder for particular social groups also predicts low trust. Similar patterns emerge for doctors: respondents who were dissatisfied with their last doctor visit are more likely to report low trust in the healthcare system, and a generally negative perception of doctors is likewise a predictor of low trust. At the country level, the analysis shows that in countries with a high CPI score (low levels of corruption in the public sector), fewer people have low trust in the healthcare system.

The two robustness checks produced comparable findings, with identical directions of association and odds ratios of similar magnitude. Differences were essentially confined to a small number of weak associations that, across the three variants, either narrowly reached statistical significance or narrowly failed to do so (supplementary Tables 2 and 3).

## Discussion

The aim of this study was to replicate and extend previous research on predictors of low trust in national healthcare systems. The ISSP 2021 data reveal several such predictors, which are compared with earlier findings below.

This study confirms substantial between-country differences in trust in the healthcare system. The previous ISSP 2011 data had already shown particularly high trust levels in Scandinavian countries [24]. This pattern is consistent with other research indicating that generalized trust is higher in these countries than elsewhere [42], to the point that trust has been described as the “Nordic gold” [26] because it supports better societal functioning. This suggests that Scandinavian healthcare systems may serve as models and that further research is needed on what makes these systems appear trustworthy. One possible explanation is that perceived corruption is low in these countries, which explains part of the variation in trust. However, it is admittedly difficult to disentangle cause and effect when it comes to corruption and trust [43].

In the present analysis, younger age is associated with lower trust, which is consistent with Zhao et al. [22] but contradicts Lamot et al. [23]. Trust is also lower among women than among men, in line with the international study by Kruk et al. [10] and in contrast to findings from China by Chen and Cheng [20]. Educational differences are less straightforward. In model 3 that includes only sociodemographic variables, lower educational attainment is associated with lower trust, consistent with previous research [10]. However, this association disappears after adjusting for health status and reverses once attitudes and experiences are added. This pattern is also reported in [24] and suggests that the initially lower trust among respondents with less education may largely reflect their poorer average health, less favourable experiences, and more negative attitudes.

Regarding health status, this analysis confirms that poor self-rated health predicts low trust in the healthcare system [4, 21, 22, 44]. This is problematic because low self-rated health is associated with higher morbidity [45, 46] and higher mortality [47]. What might be problematic here is that individuals with low trust in the healthcare system are less likely to use it [2, 48]. This carries the risk that reduced use will lead to a downward spiral resulting in poorer health. From a political science perspective, there is a risk that poor health goes hand in hand with lower levels of political engagement, meaning that there is less incentive for health policy-makers to advocate for these people [49]. Mattila and Rapeli have identified a link between poor health and low trust in politics, based on data from the European Social Survey [44]. They explain this theoretically using the psychological-democratic contract theory. According to this theory, illness can lead those affected to perceive an imbalance between their expectations of health policy and the actual outcomes it delivers. Compared with self-rated health, the presence of a chronic condition is not a strong predictor; indeed, the association is not significant in Model 4, and individuals without chronic conditions tend to have slightly less trust in the healthcare system. Beyond trust, this confirms the important role of self-rated health as a predictor in public health research, as evidenced by such diverse outcomes as life satisfaction [50], medicine use [51], and dementia [52].

Consistent with earlier studies, this analysis shows that trust in the healthcare system is low among vaccine sceptics and opponents [14, 23]. In model 4, respondents who did not believe alternative medicine to be superior were less likely to have low levels of trust, which is consistent with the findings of Lamot et al. [23]. However, when attitudes and experiences with doctors and barriers to access are taken into account in model 5, this association disappears, in contrast to the structural equation model by Lamot et al., which considers only political attitudes and attitudes towards alternative medicine as predictors of trust. Overall, this suggests that these relationships need to be better substantiated theoretically and examined empirically, particularly as other studies have treated trust in the healthcare system as an independent variable and willingness to be vaccinated as an outcome [13, 14]. The pattern for voting behaviour also adds nuance. Lamot et al.’s structural equation model found a negative linear relationship between political orientation (from left to right) and trust in the healthcare system. When voters are grouped as in the present study, however, trust is lower among voters on the extreme left and extreme right, as well as among non-voters, compared with centrist voters. This aligns with a Dutch study showing that political trust is higher among centrist voters than among people with extreme left-wing or right-wing views [53].

Well-established associations are also confirmed with respect to doctors and access barriers. Respondents dissatisfied with their last medical treatment report lower trust in the healthcare system, as shown in both recent work [54] and earlier ISSP analyses [22, 24]. Beyond personal experience, the overall evaluation of the medical profession is an independent predictor of trust, confirming previous studies [7, 18, 54]. A similar pattern is observed for access barriers: both personal experience of barriers [10, 54] and the general perception of unequal access [20] independently predict low trust.

Taken together, these findings indicate that trust in the healthcare system has multiple predictors, suggesting several potential levers for strengthening trust. These operate at different levels, ranging from behaviour in direct doctor–patient interactions to the broader public image of physicians and the healthcare system. At the same time, predictors such as perceived corruption in the country of residence, voting behaviour, and attitudes toward vaccination indicate that trust in the healthcare system is shaped not only by factors within the health sector, but also reflects the wider social capital of a society.

### Limitations and future directions

A key limitation is that the ISSP is a cross-sectional survey, so causal relationships cannot be established. In addition, participating countries differ in their sampling designs and survey modes, meaning that cross-national differences in responses may partly reflect methodological variation. Differences in selection probabilities are addressed through weighting, and the impact of differing survey approaches has been examined in detail and is often small [55, 56]. Nonetheless, greater standardisation would be desirable, for example regarding upper age limits—which in some cases exclude substantial parts of the population—and decisions about whether to include only citizens or also foreign residents.

The survey was also carried out at different points during and shortly after the COVID-19 pandemic, a period when healthcare systems were under particular public scrutiny. However, analyses of the previous ISSP Health module from 2011 do not suggest that the basic associations differ fundamentally [24, 57].

A major strength of the ISSP is its large number of participating countries and nationally representative samples. Moreover, the ISSP provides a broad set of variables. This breadth, however, necessarily limits depth; many concepts are measured with single items. For a construct such as trust, more frequent use of validated multi-item instruments would be beneficial, and methodology reports should more systematically link measures to underlying scientific concepts, as recommended by Taylor et al. [3]. One strength of the analysis is that the results do not change substantially when the outcome is analysed using ordered logistic regression or when non-substantive responses are excluded.

Future research should better clarify the causal directions underlying the observed associations, which calls for longitudinal study designs. It would also be important to examine more closely the different levels at which causes operate, and how these levels interact—for example, the interplay between individual characteristics, the immediate social context, and the healthcare system as a whole.

## Conclusion

Trust in national healthcare systems emerges as a multidimensional construct shaped by a wide range of individual characteristics, attitudes, and lived experiences. The study’s findings underscore that health status—particularly poor self-rated health—is a central predictor of low trust, reinforcing concerns that those most in need of care may simultaneously feel least confident in the system designed to support them. Political attitudes also play an important role: individuals with extreme political orientations or sceptical views toward vaccination exhibit significantly lower trust, highlighting how broader sociopolitical dynamics are correlated with perceptions of healthcare institutions.

Equally critical are people’s interactions with the healthcare system. Negative experiences with medical professionals, dissatisfaction with recent treatment, and perceptions of unequal or difficult access correlate with low trust. These patterns suggest that trust-building requires efforts not only at the system level but also within everyday clinical encounters. Improving communication, strengthening patient–physician relationships, and ensuring respectful, responsive care are essential levers.

At the structural level, reducing barriers to access and addressing perceived inequalities remain vital. Enhancing transparency, ensuring fairness, and investing in equitable healthcare delivery can build trust. Taken together, these findings point to multiple actionable pathways for strengthening public trust—a foundational component of effective and sustainable healthcare systems.

## Supplementary Information

Below is the link to the electronic supplementary material.

Supplementary Material 1

## Data Availability

The datasets analysed during the current study are available in the GESIS repository: International Social Survey Programme: Health II – ISSP 2021; 10.4232/5.ZA8000.2.0.0.

## Abbreviations

AIC: Akaike Information Criterion
BIC: Bayesian Information Criterion
CPI: Corruption Perceptions Index
ISSP: International Social Survey Programme
OR: Odds Ratio
Ref: Reference group
SE: Standard error
