# Supplementary material for: Predictors of low trust in national healthcare systems in 30 countries
Source: BMC Health Serv Res. 2026 Jun 29;26:885. doi: 10.1186/s12913-026-15026-8 (PMC13317424; doi:10.1186/s12913-026-15026-8)
Supplement: Supplementary file 1 — Supplementary Material 1 [file 12913_2026_15026_MOESM1_ESM.docx]

# Predictors of low Trust in national Healthcare Systems in 30 Countries (Supplement)

Andreas Heinz^1^

^1^Department of Health, Research Center for Public, Planetary and Digital Health, IU International University of Applied Sciences, Erfurt, Germany

**Author Note**

Andreas Heinz
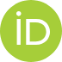
 <https://orcid.org/0000-0002-2362-417X>

Supplementary Table 1: Distribution of trust in the healthcare system by country

|  | In general, how much confidence do you have in the health care system in [country] | | | | |  |
| --- | --- | --- | --- | --- | --- | --- |
|  | Complete confidence | A great deal of confidence | Some confidence | Very little confidence | No confidence at all | n |
| AU-Australia | 5,9% | 44,3% | 40,7% | 7,4% | 1,7% | 848 |
| AT-Austria | 7,3% | 52,6% | 28,7% | 9,9% | 1,6% | 1,376 |
| CN-China | 24,8% | 47,6% | 24,6% | 2,1% | 0,8% | 2,587 |
| TW-Taiwan | 12,7% | 44,7% | 36,0% | 6,1% | 0,6% | 1,560 |
| HR-Croatia | 6,0% | 21,4% | 49,2% | 17,9% | 5,6% | 1,075 |
| CZ-Czech Republic | 18,4% | 36,9% | 33,8% | 9,8% | 1,1% | 1,230 |
| DK-Denmark | 8,1% | 59,3% | 26,4% | 5,4% | 0,8% | 1,456 |
| FI-Finland | 16,0% | 61,1% | 19,2% | 3,1% | 0,5% | 926 |
| FR-France | 3,4% | 34,9% | 52,5% | 7,7% | 1,5% | 1,424 |
| DE-Germany | 6,7% | 62,0% | 24,5% | 6,0% | 0,8% | 1,577 |
| HU-Hungary | 6,3% | 31,9% | 49,0% | 9,8% | 3,1% | 891 |
| IS-Iceland | 2,6% | 34,9% | 48,1% | 13,2% | 1,2% | 937 |
| IN-India | 15,2% | 35,6% | 33,4% | 11,9% | 3,7% | 1,506 |
| IL-Israel | 2,7% | 33,2% | 50,2% | 11,3% | 2,6% | 1,101 |
| IT-Italy | 2,9% | 30,7% | 45,6% | 15,9% | 4,9% | 1,030 |
| JP-Japan | 3,8% | 23,5% | 62,7% | 9,4% | 0,6% | 1,300 |
| MX-Mexico | 7,6% | 19,8% | 37,1% | 22,4% | 13,1% | 870 |
| NL-Netherlands | 6,6% | 64,1% | 25,9% | 2,8% | 0,7% | 1,186 |
| NZ-New Zealand | 3,3% | 31,6% | 47,4% | 13,0% | 4,8% | 1,002 |
| NO-Norway | 13,3% | 69,9% | 14,4% | 2,0% | 0,4% | 1,379 |
| PH-Philippines | 14,8% | 23,8% | 48,8% | 11,1% | 1,6% | 1,677 |
| PL-Poland | 1,7% | 10,4% | 55,1% | 23,1% | 9,7% | 1,035 |
| RU-Russia | 8,8% | 13,4% | 38,6% | 23,6% | 15,7% | 1,520 |
| SK-Slovakia | 7,5% | 30,6% | 38,5% | 16,5% | 7,0% | 968 |
| SI-Slovenia | 9,8% | 55,9% | 25,4% | 7,0% | 1,9% | 905 |
| ZA-South Africa | 12,0% | 19,2% | 37,9% | 18,1% | 12,8% | 2,610 |
| SR-Suriname | 3,7% | 10,7% | 36,6% | 39,5% | 9,5% | 1,041 |
| CH-Switzerland | 11,1% | 61,3% | 24,0% | 2,9% | 0,7% | 3,027 |
| TH-Thailand | 2,5% | 27,6% | 43,3% | 15,5% | 11,1% | 1,310 |
| US-United States | 7,4% | 19,8% | 48,4% | 20,8% | 3,6% | 1,038 |
|  |  |  |  |  |  | 40,392 |

Supplementary Table 2: Multilevel ordered logistic regression of predictors of low trust in the healthcare system

|  | Model 1: Null model with cluster effect | Model 2: Covariates added | Model 3: Health status added | Model 4: Medical scepticism and political orientation added | Model 5: Perception and experiences with doctors and access barriers and CPI added |
| --- | --- | --- | --- | --- | --- |
| **Fixed Effects** |  | OR (SE) | OR (SE) | OR (SE) | OR (SE) |
| Age |  | 0.995 (0.001)*** | 0.992 (0.001)*** | 0.995 (0.001)*** | 0.997 (0.001)*** |
| Gender (female; ref. = male) |  | 1.093 (0.019)*** | 1.074 (0.019)*** | 1.075 (0.019)*** | 1.063 (0.019)** |
| Educational level (ref. = high) |  |  |  |  |  |
| medium |  | 1.126 (0.023)*** | 1.069 (0.023)** | 0.977 (0.023) n.s. | 0.932 (0.023)** |
| low |  | 1.040 (0.028) n.s. | 0.960 (0.028) n.s. | 0.845 (0.028)*** | 0.783 (0.029)*** |
| Self rated health (ref. = very good/excellent) |  |  |  |  |  |
| good |  |  | 1.472 (0.023) *** | 1.464 (0.023)*** | 1.341 (0.023)*** |
| poor/fair |  |  | 2.170 (0.027)*** | 2.139 (0.027)*** | 1.704 (0.028)*** |
| No chronic condition (ref. = chronic condition) |  |  | 1.013 (0.023) n.s. | 0.989 (0.023) n.s. | 1.025 (0.024) n.s. |
| Alternative medicine better (ref. = agree) |  |  |  |  |  |
| neutral or no answer |  |  |  | 0.976 (0.027) n.s. | 1.113 (0.027)*** |
| disagree |  |  |  | 0.833 (0.028)*** | 1.043 (0.028) n.s. |
| Attitude towards vaccination (1 = positive; 5 = negative) |  |  |  | 1.318 (0.010)*** | 1.139 (0.010)*** |
| Vote last election (ref. = center) |  |  |  |  |  |
| no vote, no answer, other |  |  |  | 1.179 (0.034)*** | 1.133 (0.034)*** |
| Far right |  |  |  | 1.565 (0.066)*** | 1.410 (0.067)*** |
| Right, conservative |  |  |  | 0.922 (0.038)* | 0.949 (0.039) n.s. |
| Left, center |  |  |  | 0.931 (0.039) n.s. | 0.953 (0.040) n.s. |
| far left |  |  |  | 1.193 (0.063)** | 1.196 (0.064)** |
| Experienced barriers to access in the healthcare system (yes; ref. = no such experience) |  |  |  |  | 1.280 (0.024)*** |
| Perception of equality of access to healthcare in country (0 = equal; 8 = highly unequal) |  |  |  |  | 1.051 (0.006)*** |
| Perception of doctors in country (0 = positive; 5 = negative) |  |  |  |  | 2.310 (0.014)*** |
| Satisfied with last treatment by a doctor (ref. = satisfied) |  |  |  |  |  |
| neutral or no answer |  |  |  |  | 1.797 (0.028)*** |
| not satisfied |  |  |  |  | 2.578 (0.037)*** |
| CPI (0 = corrupt -100 = clean) |  |  |  |  | 0.987 (0.006)* |
| **Random effects** |  |  |  |  |  |
| Variance explained at country level (SE) | 0.677 (0.179) | 0.666 (0.176) | 0.647 (0.171) | 0.577 (0.152) | 0.430 (0.116) |
| **ICC Country** | 0.171 | 0.168 | 0.164 | 0.149 | 0.116 |
| **Model fit** |  |  |  |  |  |
| AIC | 659562,372 | 660755,913 | 667392,939 | 675744,727 | 724843,979 |
| BIC | 659570,978 | 660764,519 | 667401,545 | 675753,333 | 724852,585 |

***p < 0.001; **p < 0.01; *p < 0.05. AIC, Akaike Information Criterion; BIC, Bayesian Information Criterion; CPI, Corruption Perceptions Index; OR, Odds Ratio; ref., reference category; SE, Standard error of the log-odds; sample size: 40,392 participants from 30 countries

Supplementary Table 3: Multilevel logistic regression of predictors of low trust in the healthcare system (“no answer“ excluded)

|  | Model 1: Null model with cluster effect | Model 2: Covariates added | Model 3: Health status added | Model 4: Medical scepticism and political orientation added | Model 5: Perception and experiences with doctors and access barriers and CPI added |
| --- | --- | --- | --- | --- | --- |
| **Fixed Effects** |  | OR (SE) | OR (SE) | OR (SE) | OR (SE) |
| Age |  | 0.993 (0.001)*** | 0.989 (0.001)*** | 0.993 (0.001)*** | 0.995 (0.001)*** |
| Gender (female; ref. = male) |  | 1.088 (0.023)*** | 1.069 (0.024)*** | 1.068 (0.024)** | 1.049 (0.025)n.s. |
| Educational level (ref. = high) |  |  |  |  |  |
| medium |  | 1.244 (0.034)*** | 1.175 (0.028)*** | 1.069 (0.029)* | 1.025 (0.031)n.s. |
| low |  | 1.140 (0.028)*** | 1.046 (0.035) n.s. | 0.909 (0.036)** | 0.845 (0.038)*** |
| Self rated health (ref. = very good/excellent) |  |  |  |  |  |
| good |  |  | 1.582 (0.028) *** | 1.585 (0.029)*** | 1.463 (0.030)*** |
| poor/fair |  |  | 2.443 (0.034)*** | 2.434 (0.035)*** | 1.953 (0.037)*** |
| No chronic condition (ref. = chronic condition) |  |  | 1.036 (0.029)n.s. | 1.018 (0.030) n.s. | 1.076 (0.031)* |
| Alternative medicine better (ref. = agree) |  |  |  |  |  |
| Neutral |  |  |  | 1.047 (0.034) n.s. | 1.188 (0.036)*** |
| disagree |  |  |  | 0.806 (0.034)*** | 1.009 (0.036) n.s. |
| Attitude towards vaccination (1 = positive; 5 = negative) |  |  |  | 1.330 (0.013)*** | 1.152 (0.014)*** |
| Vote last election (ref. = center) |  |  |  |  |  |
| no vote, no answer, other |  |  |  | 1.271 (0.043)*** | 1.216 (0.045)*** |
| Far right |  |  |  | 1.561 (0.082)*** | 1.400 (0.087)*** |
| Right, conservative |  |  |  | 0.957 (0.048) n.s. | 0.969 (0.050) n.s. |
| Left, center |  |  |  | 0.931 (0.049) n.s. | 0.942 (0.052) n.s. |
| far left |  |  |  | 1.227 (0.079)** | 1.222 (0.084)* |
| Experienced barriers to access in the healthcare system (yes; ref. = no such experience) |  |  |  |  | 1.294 (0.031)*** |
| Perception of equality of access to healthcare in country (0 = equal; 8 = highly unequal) |  |  |  |  | 1.043 (0.008)*** |
| Perception of doctors in country (0 = positive; 5 = negative) |  |  |  |  | 2.315 (0.020)*** |
| Satisfied with last treatment by a doctor (ref. = satisfied) |  |  |  |  |  |
| neutral |  |  |  |  | 2.252 (0.045)*** |
| not satisfied |  |  |  |  | 2.367 (0.053)*** |
| CPI (0 = corrupt -100 = clean) |  |  |  |  | 0.985 (0.008)* |
| **Random effects** |  |  |  |  |  |
| Variance explained at country level (SE) | 0.800 (0.212) | 0.783 (0.207) | 0.780 (0.206) | 0.729 (0.193) | 0.636 (0.172) |
| **ICC Country** | 0.196 | 0.192 | 0.192 | 0.181 | 0.162 |
| **Model fit** |  |  |  |  |  |
| AIC | 163656,409 | 163881,281 | 164880,376 | 166180,630 | 172113,914 |
| BIC | 163664,890 | 163889,762 | 164888,857 | 166189,111 | 172122,395 |

***p < 0.001; **p < 0.01; *p < 0.05. AIC, Akaike Information Criterion; BIC, Bayesian Information Criterion; CPI, Corruption Perception Index; OR, Odds Ratio; ref., reference category; SE, Standard error of the log-odds; sample size: 35,651 participants from 30 countries

Supplementary Table 4: Comparison of the analytic sample with the complete dataset

| Variable | Analytic sample (n = 40,392) | Complete dataset (n = 44,549) | | |
| --- | --- | --- | --- | --- |
|  | Means (SD) or percentages | Means (SD) or percentages of valid values | Missings | comment |
| Trust in healthcare system | 9.4% complete confidence  38.8% a great deal of confidence  36.7% some confidence  11.2% very little confidence  4.0% no confidence at all | 9.4% complete confidence  38.2% a great deal of confidence  36.9% some confidence  11.4% very little confidence  4.1% no confidence at all | 662 (1.5%) |  |
| Age | Mean 49,32 (17.29 SD) | Mean 49,96 (17.46 SD) | 293 (0.7%) |  |
| Gender | 46.4% male  53.6% female | 46.2% male  53.8% female | 76 (0.2%) |  |
| Educational level (ISCED) | 38.0% high  37.7% medium  24.3% low | 37.1% high  37.4% medium  25.5% low | 680 (1.5%) |  |
| Self rated health | 35.3% very good/excellent  37.9% good  26.8% poor/fair | 34.9% very good/excellent  37.9% good  27.2% poor/fair | 449 (1.0%) |  |
| Chronic condition/disability | 30.8% yes  69.2% no | 31.0% Yes  69.0% no | 472 (1.1%) |  |
| Alternative medicine better | 19.2% agree  39.3% neutral or no answer  41.5% disagree | 20.8% agree  35.8% neither agree nor disagree  43.4% disagree | 3405 (7.6%) | missings assigned to “neutral or no answer” ^1^ |
| Attitude towards vaccination (1 = positive; 5 = negative) | Mean 2.35 (SD 1.08) | Mean 2.36 (SD 1.08) | 1313 (2.9%) |  |
| Vote last election | 3.0% far left  14.5% left, center  12.1% center  15.9% right, conservative  2.6% far right  51.8% no vote, no answer, other | 5.9% far left  28.7% left, center  23.3% center  31.0% right, conservative  5.1% far right  4.7% other  1.4% invalid ballot | 22,266 total (50.0%):  2,124 no answer  803 insufficient information  2,601 refused  8,148 not applicable  8,590 not available (CN, SR, TW, ZA) | all missings, other, and invalid ballot assigned to “no vote, no anser, other” |
| Experienced barriers to access in the healthcare system | 23.0% experienced at least one barrier  77.0% no barrier or no treatment needed | 23.0% experienced at least one barrier  77.0% no barrier or no treatment needed | 175 (0.4%) |  |
| Perception of equality of access to healthcare in country (0 = equal; 8 = highly unequal) | mean 2.90 (SD 1.81) | Mean 2.87 (SD 1.82) | 511 (1.1%) |  |
| Perception of doctors (0 = positive; 5 = negative) | mean 1.66 (SD 0.79) | 1.67 Mean (SD 0.79) | 346 (0.8%) |  |
| Satisfied with last treatment by a doctor | 76.9% satisfied  15.4% neutral or no answer  7.7% not satisfied | 81.1% satisfied  10.7% neither satisfied nor dissatisfied  8.2% not satisfied | 2,865 total (6.4%):  174 no answer  789 “can’t choose”  1,902 “does not apply” | all missings assigned to “neutral or no answer” ^1^ |

SD, standard deviation; unweighted data; ^1^ not assigned to “neutral or no answer” in *Table 3: Multilevel logistic regression of predictors of low trust in the healthcare system (“no answer“ excluded)* in this Supplement

Supplementary Figure 1: Level of low trust in the healthcare system by Corruption Perceptions Index

Source: Own calculation based on (1,2)

1. ISSP Research Group -. ZA8000 International Social Survey Programme: Health and Health Care II - ISSP 2021ZA8000 International Social Survey Programme: Health and Health Care II - ISSP 2021 [Internet]. GESIS Data Archive; 2024 [cited 2025 Sep 4]. Available from: https://search.gesis.org/research_data/ZA8000?doi=10.4232/5.ZA8000.2.0.0 doi:10.4232/5.ZA8000.2.0.0

2. Transparency International, editor. Corruptions Perceptions Index 2021 [Internet]. Berlin: Transparency International; 2022 [cited 2026 Jun 3]. Available from: https://images.transparencycdn.org/images/CPI2021_Report_EN-web.pdf
